# Barriers to cross-disciplinary knowledge flow: The case of medical education research

**DOI:** 10.1007/s40037-021-00685-6

**Published:** 2021-10-14

**Authors:** Mathieu Albert, Paula Rowland, Farah Friesen, Suzanne Laberge

**Affiliations:** 1 Wilson Centre and Department of Psychiatry, University of Toronto, University Health Network, Toronto, ON Canada; 2 Wilson Centre and Department of Occupational Science & Occupational Therapy, University of Toronto, University Health Network, Toronto, ON Canada; 3 Women’s College Hospital, Toronto, ON Canada; 4 School of Kinesiology and Physical Activity Sciences, Université de Montréal, Montréal, QC Canada

**Keywords:** Interdisciplinarity, Medical education, Higher education, Citations analysis, Doxa, Bourdieu

## Abstract

**Introduction:**

The medical education research field operates at the crossroads of two distinct academic worlds: higher education and medicine. As such, this field provides a unique opportunity to explore new forms of cross-disciplinary knowledge exchange.

**Methods:**

Cross-disciplinary knowledge flow in medical education research was examined by looking at citation patterns in the five journals with the highest impact factor in 2017. To grasp the specificities of the knowledge flow in medical education, the field of higher education was used as a comparator. In total, 2031 citations from 64 medical education and 41 higher education articles published in 2017 were examined.

**Results:**

Medical education researchers draw on a narrower range of knowledge communities than their peers in higher education. Medical education researchers predominantly cite articles published in health and medical education journals (80% of all citations), and to a lesser extent, articles published in education and social science journals. In higher education, while the largest share of the cited literature is internal to the domain (36%), researchers cite literature from across the social science spectrum. Findings suggest that higher education scholars engage in conversations with academics from a broader range of communities and perspectives than their medical education colleagues.

**Discussion:**

Using Pierre Bourdieu’s concepts of doxa and field, it is argued that the variety of epistemic cultures entering the higher education research space contributes to its interdisciplinary nature. Conversely, the existence of a relatively homogeneous epistemic culture in medicine potentially impedes cross-disciplinary knowledge exchange.

**Supplementary Information:**

The online version of this article (10.1007/s40037-021-00685-6) contains supplementary material, which is available to authorized users.

## Introduction

A growing number of researchers have been studying interdisciplinary knowledge flow in recent decades [1–4], contributing to the debate around the volume of knowledge exported from, and imported by disciplines. In contrast to the common belief according to which disciplines are silos [5, 6], these researchers [1–4] offer a more nuanced perspective. While some disciplines, such as economics, are relatively insular, others, such as demography and geography, are largely open to external knowledge [1]. We contribute to this literature by focusing on medical education research. Medical education represents a unique field for examining cross-disciplinary knowledge circulation since it operates at the crossroads of two distinct academic worlds: higher education and medicine. Because of this position, medical education is one of the few research areas where the medical and the social sciences/education epistemic cultures meet, generating opportunities for new forms of cross-disciplinary knowledge exchange. Studying the knowledge flow in medical education will provide members of the field with empirical data on the current knowledge landscape in their domain. This field-level data may help identify potential knowledge gaps, weaknesses, and strengths relevant to medical education research.

The questions at the core of our research are: Do medical education researchers draw on knowledge developed in education, higher education, and other education-related disciplines (e.g., sociology, psychology, political sciences, economics)? Do they participate in the knowledge flow across the social sciences related to education or do they focus on alternative pools of knowledge more closely aligned with medical research? We explore these interrelated questions by examining knowledge flow in medical education research by analyzing the references of publications, which is known as citation analysis.

Building on a preliminary study [7], we use a comparative design to assess the level of cross-disciplinary communication in medical education research in comparison with higher education research.

The adoption of a comparative strategy enabled us to contextualize our findings by putting them side by side with those of a cognate field [2]. We chose higher education as a comparator because it shares several features with medical education research. Both are sub-domains of education and focus on educational issues at the post-secondary level. Both also have a topical rather than a disciplinary focus, and comprise an applied dimension seeking to inform practice. Further, medicine and higher education are large social institutions and as such call for a broad range of disciplines to fully grasp their connections with social actors and organizations (e.g., professional regulatory bodies, political and economic actors, private and public funding agencies), and to understand how these connections may shape their learning activities and curricular design.

## Theoretical framework

To understand the logic underpinning the citation patterns in medical education, we draw on Pierre Bourdieu’s concepts of doxa [8] and field [9]. For Bourdieu, doxa is “a set of fundamental beliefs which does not even need to be asserted in the form of an explicit, self-conscious dogma” [8, p. 16]. Doxa operates as the cultural orthodoxy of a field and, as such, delineates the unspoken but acknowledged rules of the game therein. Members of a scientific field (e.g., epidemiology, sociology, education) share a set of assumptions regarding essential aspects of academic practice: legitimate methodologies, research productivity, journal rankings, etc. Players promoting heterodox (e.g., diverging, nonconforming) views run the risk of being marginalized as their positions may be considered discordant [10, 11].

For Bourdieu, a field is a space in which social actors struggle for scientific authority, which is understood as the capacity to define what legitimate science is, and to set the rules of the game. Scientists engage in struggles with their peers to have their own practices perceived as legitimate—and to become the new doxa. Scientists who gain scientific authority are those who succeed in having their views on science and research practices perceived as the legitimate way of thinking about and engaging in science. The features of their work (the methods they use, the journals in which they publish, the scholars they cite, etc.) become understood as the legitimate features of ‘good’ practices and the standard against which others’ work is judged [9].

## Methods

We performed a bibliometric analysis of the reference lists of publications from medical education and higher education journals. Building on the recent work of Jacobs [12] comparing knowledge flow in education and psychology, we examine the flow of ideas and knowledge entering medical education and higher education from external disciplines and research areas. A detailed description of the methods can be found in the online Appendix (Electronic Supplementary Material).

First, we identified five medical education and five higher education journals with the highest impact factor by using the 2017 Journal Citation Reports (JCR). For medical education these were: *Academic Medicine, Medical Education, Advances in Health Sciences Education, Medical Teacher*, and *BMC Medical Education* (Tab. 1). The five journals in higher education were: *Studies in Higher Education, Journal of Higher Education, Active Learning in Higher Education, Higher Education Research & Development, and Higher Education *(Tab. 2). Next, we identified articles published within these 10 journals. We targeted research articles only and excluded non-primary research formats.

**Table 1:** The five most cited journals in medical education research in 2017

Medical Education Journals	Total research articles published in 2017	10% of research articles published in 2017
Academic Medicine (JIF: 4.8)	134	13
Medical Education (JIF: 4.4)	81	8
Advances in Health Sciences Education (JIF: 2.5)	66	7
Medical Teacher (JIF: 2.4)	124	12
BMC Medical Education (JIF: 1.5)	242	24
Total	647	64

Source: Selected JCR Year: 2017 Selected Editions: SCIE, SSCI Selected Categories: “EDUCATION & EDUCATIONAL RESEARCH”,“EDUCATION, SCIENTIFIC DISCIPLINES” Selected Category Scheme: WoS

*JIF* Journal impact factor

**Table 2:** The five most cited journals in higher education research in 2017

Higher Education Journals	Total research articles published in 2017	10% of published research articles in 2017
Studies in Higher Education (JIF: 2.3)	138	14
Journal of Higher Education (JIF: 2.2)	35	4
Higher Education Research & Development (JIF: 2.0)	97	10
Active Learning in Higher Education (JIF: 1.9)	17	2
Higher Education (JIF: 1.9)	112	11
Total	399	41

Selected JCR Year: 2017 Selected Editions: SCIE, SSCI Selected Categories:“EDUCATION & EDUCATIONAL RESEARCH”,“EDUCATION, SCIENTIFIC DISCIPLINES” Selected Category Scheme: WoS

*JIF* Journal impact factor

The total number of research articles published in the 10 targeted journals in 2017 was 1046 (647 in medical education; 399 in higher education). We randomly selected 10% of these articles, which totalled 105 articles (64 medical education; 41 higher education). These articles cited a total of 1959 articles: 1412 in the 64 medical education sampled articles, 547 in the 41 higher education sampled articles.

**Fig. 1:**
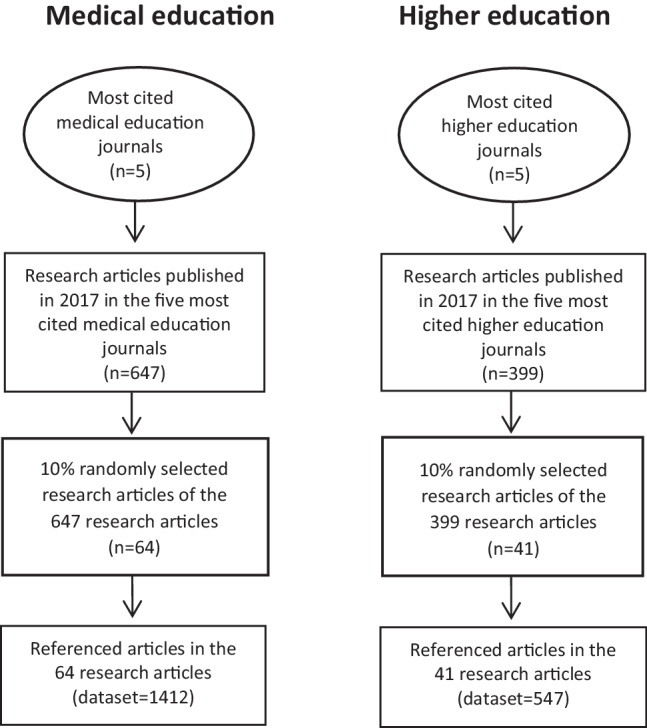
Procedure used to construct the dataset of referenced articles from the five most cited medical education journals and the five most cited higher education journals

The procedure used to construct the dataset of referenced articles from the five most cited medical education journals and the five most cited higher education journals is shown in Fig. 1.

The same sampling procedure was used for cited books and book chapters. The 64 medical education research articles referenced 154 books and 57 books chapters; 10% represent 15 books and six book chapters. The 41 higher education research articles referenced 373 books and 138 book chapters; 10% represent 37 books and 14 book chapters. In addition to the 1959 referenced articles, we included these 52 books and 20 book chapters for a total of 2031 references in our dataset. See the Appendix of the Electronic Supplementary Material for a table outlining the distribution of research articles, books, and book chapters included in our dataset.

To identify which disciplines and research areas medical education and higher education researchers draw from, we inductively developed a typology of eight knowledge orientations, which we labelled knowledge clusters. These knowledge clusters were: 1) Medical Education; 2) Applied Health (mostly health services research/clinical research); 3) Interdisciplinary Health; 4) Disciplinary/Institutionalized Research Areas; 5) Education; 6) Topic-Centred (non-health); 7) Profession/Science Education; and 8) Higher Education. The interdisciplinary expertise of our research team (sociology, organizational studies, information sciences, kinesiology, and anthropology) proved to be useful for coding the references. In order to provide as much detail as possible on our categorization, we include a table in the Appendix of the Electronic Supplementary Material listing the three journals with the highest number of citations for each cluster as well as one book and/or book chapter.

## Results

Citation patterns suggest that medical education researchers predominantly draw on research published within their own field (40% of references); and research published in clinical and health services research journals (also 40% of references) (Fig S1 of the Electronic Supplementary Material). Knowledge emanating from other sources occupies a relatively small portion of the medical education intellectual space: Interdisciplinary Health Research 8%; Disciplines and Institutionalized Research Areas 6%; Topic Centred research and Education 3%.

These patterns demonstrate that medical education scholars may be studying education at the post-secondary level in medicine, but they do so using a vastly different knowledge repertoire than the one used in higher education (Fig S2 of the Electronic Supplementary Material) and by the education research community more broadly [13, 14]. Their primary source of external knowledge is not the education-related social sciences, such as sociology, psychology, economics, political sciences, but applied knowledge published in clinical and health services research journals. Instead of relating themselves to the higher education community, medical education researchers draw primarily on the knowledge originating from their medical work contexts, faculties of medicine.

The distinctiveness of the knowledge flow in medical education stands out clearly when compared with the citation patterns in higher education. While proportionally the largest share of the literature consulted by higher education scholars is unsurprisingly from their own field (36%), the knowledge drawn from outside their domain is spread across various intellectual horizons. Disciplinary and Institutionalized Research Areas represent 26% of the external knowledge cited by higher education researchers; Education 19%; Topic-Centred research 15%; and Profession and Science Education 3% (Fig S2 of the Electronic Supplementary Material). These patterns suggest that higher education scholars engage in conversations with academics from a broader range of communities and perspectives than their medical education colleagues and that no form of inquiry overshadows other forms as the distribution is more equally distributed across clusters.

### Disciplinary and institutionalized research areas

Within the Disciplinary and Institutionalized Research Areas cluster, the academic space is characterized by a sharp imbalance of knowledge inputs in medical education. Psychology occupies 67% of the space, while the other six disciplines and research domains share the remaining 33% (Fig S3 of the Electronic Supplementary Material). The contribution of each of these knowledge areas is consequently marginal, oscillating between 12% of the disciplinary space (sociology and anthropology) and 3% (biology), with business, management and organizational studies (7%), social psychology (4%), and humanities and philosophy (4%) sitting in between.

The disciplinary imbalance in medical education is made more apparent when we compare it to the patterns of disciplinary representation in higher education (Fig S4 of the Electronic Supplementary Material). If the number of disciplines in higher education is not significantly higher than in medical education (9 in higher education versus 7 in medical education), their weight is more evenly distributed, suggesting inputs from a broader range of disciplines. Higher education scholars draw relatively equally from economics (25%) and political science (24%), slightly less, but still equally, from psychology (15%) and sociology/anthropology (14%), and to a lesser extent from five other knowledge clusters. The higher education scholars seem to be engaging with ideas and concepts from a broader variety of social sciences fields than their medical education colleagues.

### University affiliation and academic degree

How can we explain the different citation patterns between the fields of higher education and medical education? One way is to examine the university affiliation and academic degree of the first author of the 105 articles in our sample. These features can provide insights on the research culture internalized by the authors during their training years and their current academic culture. As member of an “academic tribe” [15], academics’ research practices, including what they read and cite, are inevitably shaped by the disciplinary culture of the group they evolved within.

Nearly 90% (*n* = 57) of medical education authors were appointed within a faculty of medicine. The remaining authors hailed from psychology (*n* = 2), kinesiology (*n* = 2), education (*n* = 1), anthropology (*n* = 1), and a private research firm (*n* = 1). Close to 60% of included authors hold MD degrees (*n* = 37), some with a PhD (*n* = 11). The other 40% are divided between non-health-related PhDs (*n* = 18), masters (*n* = 2), and individuals holding a professional degree either in nursing, pharmacy, or dietetics (*n* = 5) (Fig S5 of the Electronic Supplementary Material).

Academic appointments for higher education authors display a different pattern. The first authors of the higher education articles are distributed across a variety of academic units (Fig S6 of the Electronic Supplementary Material). Only over one third of them are appointed to an education or higher education unit. The others are distributed across nine different categories of academic units. All first authors hold a PhD, and none report professional degrees.

## Discussion

With 80% of the references coming from within medical education or clinical and health services research journals, medical education researchers appear to be relatively inwardly focused and selective about the sources of knowledge they allow entry into their academic space. One citation pattern shows that medical education researchers have a strong connection with academics from the applied health sciences domain, from whom they garner most of their external knowledge. The second pattern shows that this external body of knowledge is mostly complemented by work internally produced by medical education scholars. Combined together, these two sources of knowledge represent most of all ideas, concepts, and empirical findings circulating within the medical education field, leaving only 20% to other academic domains (e.g., education, higher education, sociology, psychology). These trends contrast with the field of higher education and with recent bibliometric studies showing that disciplines such as anthropology, psychology, and political sciences display an external citation rate averaging between 50% and 60% [1].

Based on these findings, one could argue that interdisciplinarity for medical education researchers primarily means being inspired by, and drawing on, clinical and health services research. They therefore arguably align themselves with, and contribute to, the reproduction of the epistemic doxa of the medical sciences. Education, higher education, and other social science disciplines are granted limited access to the medical education field and are unlikely to substantially influence its research orientation. It follows that members of the field mostly inhabit an academic space somewhat removed from the debates occurring across the disciplines outside the health domain.

Our findings align with Ten Cate’s analysis [16] of the health professional education field (HPE). In a recent paper, he notes that the field seems to be relatively insular: “very little about health professions education is published in journals of the social sciences. It shows how HPE scholars may be less inclined to read and publish in these journals, and how readers of these journal may be less interested in HPE” [16, p. 7]. He concludes that the field has developed internally, “but integration with other disciplines has been limited” [16, p. 7].

How can we explain the strong alignment of the medical education field with applied health research and its low rates of knowledge exchange with the academic disciplines traditionally associated with higher education/education research? Several impeding forces might potentially act as barriers.

A first barrier is cultural and relates to the dominant epistemic doxa in medical settings. Anthropologists and sociologists have repeatedly noted an epistemic gap between social and medical researchers [10, 17–19]. This epistemic gap has been shown to be related to the scientific training customarily offered in medical schools. Throughout their training, medical students are taught that various forms of scientific endeavours are ranked according to the quality of evidence they generate [22, 23]. The randomized controlled trial method (RCT) occupies the top ranking. This method is usually considered the ‘gold standard’ as it is perceived to be the least bias-prone form of investigation. Descriptive and observational research (which includes a large portion of the social sciences) are ranked as low forms of research because of their inability to satisfactorily control for biases. As Eakin summarized: “the scientific value and superiority accorded to the RCT approach is seen as residing in its highly controlled experimental design, objective quantitative measurement, and […] in random sample selection to reduce the possibility of systematic biases” [17, p. 110]. Our aim is not to take position for or against RCTs, but rather to stress that through the learning of RCT as ‘gold standard,’ medical students acquire a hierarchized view on scientific knowledge production. This view is perpetuated and reinforced through daily interactions with other MDs and operates as a common doxa.

It becomes understandable therefore that MDs might lack familiarity with education and other social sciences literature. This literature is undiscoverable through their regular knowledge networks (e.g., PubMed) and, as one surgeon commented, is “almost impenetrable” [24, p. 514]. Learning a discipline implies the internalization of its specific codes, traditions, debates, and rules of evidence [9, 15]. How would an academic MD find the time—between clinic, teaching, and research—to engage in such a challenging journey? Why would a MD make such an attempt and unsettle their RCT-based epistemic posture when there is no incentive or benefit for doing so? The hierarchized view of science that MDs acquired throughout their training combined with the absence of exposure to education and disciplinary social sciences [19] appears to be one contributing factor to the interdisciplinary knowledge deficit in medical education we have highlighted in our study. It is worth stressing, however, that some MDs do engage with education and social sciences literature, but these are the exception, not the norm [25].

The second barrier comes from the dominant evaluation criteria used by medical schools to assess the research productivity of their faculty members (MDs and PhDs). Medical schools often value one metric: a high volume of articles published in high impact clinical journals (easily up to five or six articles per year [11]). Other forms of knowledge production are generally disregarded [26]. In this context, reading, understanding, and appropriately applying the work of scholars such as Bourdieu, Dewey, or Latour requires a protracted commitment usually out of reach to MDs aiming to meet the metrics of success in their institutional context.

The high productivity criterion may also deter MDs and PhDs from publishing books, book chapters, and lengthier articles in social sciences/higher education journals as these types of publications slow down the publication pace. These types of publications also depart from the orthodox publication scheme in medical schools and, for that reason, are granted low, if any academic value [26, 27]. As a result, the main publication option for medical education researchers is clinical journals and similar applied health-oriented journals. With their 3000-word limit however, and targeted readership (clinicians), these journals are suboptimal vehicles to develop a sophisticated analysis of educational issues and to engage in social scientific debates [20].

Medical education journals qualify as a viable option for medical education scholars located in medical schools as they sit halfway between clinical journals and higher education/education journals. While most leading journals in medical education have a word count similar or slightly higher than clinical journals, some welcome submissions with higher word counts. Scholars can thus engage in more in-depth analysis than what is possible in clinical journals without compromising their capacity to meet their medical schools’ productivity metrics [20]. Despite the fact that medical education journals have less limitations than clinical journals, medical education researchers still lack engagement with disciplinary knowledge. Therefore, while there are avenues for publication, the dominant health-oriented epistemic doxa holds.

As previous studies have suggested [10, 11, 17, 18, 21, 26], this article shows that the integration of the education/social sciences in medical schools is likely to remain challenging if they do not provide greater flexibility in their evaluation criteria. To do so, one avenue could be to perform “cognitive contextualization” [28]. Cognitive contextualization occurs when assessors suspend their own methodological preferences and views on knowledge production and conduct their assessment “according to the epistemological and methodological standards that prevail in the discipline” of the individual under evaluation [28, p. 132]. It presumes that the assessor acknowledges that different research methods and forms of outputs are equally valuable, and that the evaluation criteria used are anchored in the disciplinary standards of the scholar’s work [28].

The adoption of a cognitive contextualization approach in medical schools would allow medical education researchers to venture more confidently beyond the health research literature and take the time necessary to integrate knowledge from a broader set of disciplines into their own research. It would also allow them to develop a publication profile spanning the full range of outputs normally associated with higher education/education research. This expanded profile could in turn facilitate the establishment of connections with their higher education/education peers and increase visibility of medical education in these fields.

Another avenue worth exploring to increase cross-disciplinary exchange between medical education and higher education research could be to hire or cross-appoint medical education scholars in higher education/education departments. These scholars would operate according to the standards in these departments and would more easily connect with their peers.

As we saw earlier, in contrast to their medical education colleagues, the higher education scholars in our sample do not work in a single research environment and therefore are not exposed to only one academic doxa. They evolve in a variety of fields, each with their own standards, norms, and evaluative cultures—with overlaps between them. This institutional diversity may be part of the reason why the higher education field appears to be the locus of a notable interdisciplinary knowledge flow. This level of diversity has not yet been reached by the medical education research field, potentially because most medical education scholars operate within a single epistemic culture—one that is also reputed to be fairly rigid [8, 14, 15].

On a broader scale, our study suggests that a research field is not inherently interdisciplinary and does not integrate multiple knowledge sources by the sole virtue of being a topic-centred domain. Certain cultural and institutional conditions must be in place for cross-disciplinary communication to flourish. Without these conditions, researchers might seek to fit within the dominant doxa operating in their institution. Consequently, they might tend to align with standards that may keep them away from research communities with which interactions would be intellectually beneficial.

Our study is not without limitations. Our research focused on a single year (2017), and on journals with the highest impact factor. Therefore, our dataset does not allow us to say anything about the growth and transformation of the medical education field over the last few decades. We have taken a static photo of an otherwise rapidly evolving research domain. Further, we do not know whether new research areas are developing in mid or low impact journals and, if so, whether they draw on knowledge from outside medical education and health research. Another limitation is that we focused on research papers only. The methodological intention was to provide an overview of primary research while also ensuring appropriate comparisons across the two fields. This decision necessarily excluded other types of publication in medical education, for example review articles, methodological articles, reflection articles, and commentaries. Therefore, we do not know whether these publications draw more on interdisciplinary knowledge than research articles do and, if so, to what degree.

Future research should examine whether the forces impeding the development of an interdisciplinary medical education research field are specific to the medical environment or if they are also at work in other professions such as business, engineering, and law. Such examination would expand the comparative perspective adopted in this study and refine our understanding of the cultural and institutional factors encouraging cross-disciplinary communication or, conversely, fostering insularity.

## Supplementary Information

Appendix

Fig. S1 Distribution of medical education research citations (*n* = 1,433) per research cluster. Data presented in percentage

Fig. S2 Distribution of higher education research citations (*n* = 596) per research cluster. Data presented in percentage

Fig. S3 Disciplinary breakdown (*n* = 92) for medical education research. Data presented in percentage

Fig. S4 Disciplinary breakdown (*n* = 154) for higher education research. Data presented in percentage

Fig. S5 Academic unit appointment of 1st author of the selected articles in medical education journals (*n* = 64). Data presented in percentage

Fig. S6 Academic unit of 1st author of the selected articles in higher education (*n* = 41). Data presented in percentage

## References

[1] Truc A, Santerre O, Gingras Y, et al. The interdisciplinarity of economics.. https://papers.ssrn.com/sol3/papers.cfm?abstract_id=3669335. Accessed 8 July 2021.

[2] Porter AA, Rafols I (2009). Is science becoming more interdisciplinary? Measuring and mapping six research fields over time. Scientometrics.

[3] Larivière V, Gingras Y, Sugimoto CCCR (2014). Measuring interdisciplinarity. Beyond bibliometrics: harnessing multidimensional indicators of scholarly impact.

[4] Chen S, Arsenault C, Gingras Y (2015). Exploring the interdisciplinary evolution of a discipline: the case of biochemistry and molecular biology. Scientometrics.

[5] Newell WH (2001). A theory of interdisciplinary studies. Issues Integr Stud.

[6] Szostak R, Repko AF, Newell WH, Szostak R (2012). The interdisciplinary research process. Case studies in interdisciplinary research.

[7] Albert M, Rowland P, Friesen F (2020). Interdisciplinarity in medical education research: myth and reality. Adv Health Sci Educ.

[8] Bourdieu P (2000). Pascalian meditations.

[9] Bourdieu P (2004). Science of science and reflexivity.

[10] Albert M, Paradis E, Kuper A (2015). Interdisciplinary promises versus practices in medicine: the decoupled experiences of social sciences and humanities scholars. Soc Sci Med.

[11] Albert M, Paradis E, Kuper A, Frickel S, Albert M, Prainsack B (2017). Interdisciplinary fantasy: social scientists and humanities scholars working in faculties of medicine. Investigating interdisciplinary collaboration: theory and practice across disciplines.

[12] Jacobs JA (2014). In defense of disciplines Interdisciplinarity and specialization in the research university.

[13] Bridges D (2017). Philosophy in educational research. Epistemology, ethics, politics and quality.

[14] Furlong J (2013). Education. An anatomy of the discipline.

[15] Becher T, Trowler PR (2001). Academic tribes and territories.

[16] Cate OT (2021). Health professions education scholarship: the emergence, current status, and future of a discipline in its own right. FASEB Bioadvances..

[17] Eakin JM (2016). Educating critical qualitative health researchers in the land of the randomized controlled trial. Qual Enq.

[18] Greenhalgh T, Annandale E, Ashcroft R (2016). An open letter to The BMJ editors on qualitative research. BMJ.

[19] Albert M, Laberge S, Hodges BD (2008). Biomedical scientists’ perception of social science in health research. Soc Sci Med.

[20] Kontos P, Grigorovich A (2018). “Sleight of hand” or “selling our soul”? Surviving and thriving as critical qualitative health researchers in a positivist world. Forum Qual Soc Res.

[21] Mykhalovskiy E, Choinière J, Armstrong P (2020). Health matters. Evidence, critical social science, and health care in Canada.

[22] Goldenberg MJ (2006). On evidence-based medicine: lessons from the philosophy of science. Soc Sci Med.

[23] Timmermans S, Berg M (2003). The gold standard: the challenge of evidence-based medicine and standardization in health care.

[24] Kneebone R (2002). Total internal reflection: an essay on paradigms. Med Educ.

[25] Albert M, Laberge S, Hodges BD (2009). Boundary-work in the health research field: biomedical and clinician scientists’ perceptions of social science research. Minerva.

[26] Webster F, Gastaldo D, Durant S (2019). Doing science differently: a framework for assessing the careers of qualitative scholars in the health sciences. Int J Qual Methods.

[27] Albert M, Laberge S, McGuire W (2012). Criteria for assessing quality in academic research: the views of biomedical scientists, clinical scientists and social scientists. High Educ.

[28] Lamont M (2009). How professors think: inside the curious world of academic judgment.

