# Supplementary material for: Barriers to cross-disciplinary knowledge flow: The case of medical education research
Source: Perspect Med Educ. 2021 Oct 14;11(3):149–55. doi: 10.1007/s40037-021-00685-6 (PMC9240116; doi:10.1007/s40037-021-00685-6)
Supplement: Supplementary file 1 — Appendix [file 40037_2021_685_MOESM1_ESM.pdf]

Appendix
Detailed methods

One effective method to investigate the sources of knowledge used by medical education and higher education researchers is to perform a bibliometric analysis of the publications they cite in their published work. Building on the recent work of Jacobs [1] comparing knowledge flow in Education and Psychology, we examine the flow of ideas and knowledge entering medical education and higher education from external disciplines and research areas.

Sampling rationale and procedure for journals and research articles

We decided not to use any extant index, such as the Rao-Stirling diversity index [2], to measure the intensity of interdisciplinary knowledge exchange in medical education and higher education, nor use any existing disciplinary categorizations, such as the U.S. National Science Foundation (NSF) categorization (2006), to create our disciplinary clusters. The reasons for this are that the level of statistical sophistication provided by an existing index is unnecessary in the context of our research which remains essentially descriptive. Our intention was to anchor our categorization in the data generated by our study as it appears to us to be the most effective way for answering our specific research question, i.e., what is the status of the disciplines, of higher education/education knowledge, and of other social sciences in medical education research? A pre-formatted and generic grid could have imposed unproductive categorizations upon our analysis.

The first step in our bibliometric analysis was identifying the five medical education and five higher education journals with the highest impact factor by using the 2017 Journal Citation Reports (JCR). The JCR categories used were ‘Education & Educational Research’ and ‘Education, Scientific Disciplines’, which are where medical education and higher education journals are classified by Clarivate Analytics. The five journals with the highest impact factor in medical education (at the time we conducted our research) were: *Academic Medicine*, *Medical Education*, *Advances in Health Sciences Education*, *Medical Teacher*, and *BMC Medical Education* (Table S1). The five journals with the highest impact factor in higher education were: *Studies in Higher education*, *Journal of Higher Education*, *Active Learning in Higher Education*, *Higher Education Research & Development*, *and Higher Education* (Table S2). Journal impact factors and other forms of metrics such as the h-index have been widely criticized [3]. We do not use any of these metrics in this paper to assess journals. We use the impact factors as an indicator of the status of the journals within their community.

Table S1 The five most cited journals in medical education research in 2017

Medical Education Journals	Total research articles published in 2017	10% of research articles published in 2017
Academic Medicine (JIF*: 4.8)	134	13
Medical Education (JIF: 4.4)	81	8
Advances in Health Sciences Education (JIF: 2.5)	66	7
Medical Teacher (JIF: 2.4)	124	12
BMC Medical Education (JIF: 1.5)	242	24

Total	647	64

Source: Selected JCR Year: 2017 Selected Editions: SCIE, SSCI Selected Categories: 'EDUCATION & EDUCATIONAL RESEARCH','EDUCATION, SCIENTIFIC DISCIPLINES' Selected Category Scheme: WoS
*JIF = Journal impact factor

Table S2 The five most cited journals in higher education research in 2017

Higher Education Journals	Total research articles published in 2017	10% of published research articles in 2017
Studies in Higher Education (JIF*: 2.3)	138	14
Journal of Higher Education (JIF: 2.2)	35	4
Higher Education Research & Development (JIF: 2.0)	97	10
Active Learning in Higher Education (JIF: 1. 9)	17	2
Higher Education (JIF: 1.9)	112	11
Total	399	41

Selected JCR Year: 2017 Selected Editions: SCIE, SSCI Selected Categories: 'EDUCATION & EDUCATIONAL RESEARCH','EDUCATION, SCIENTIFIC DISCIPLINES' Selected Category Scheme: WoS
*JIF = Journal impact factor

In selecting the five journals with the highest impact factors in each of the two targeted domains, our goal was not to create a sample of articles representative of all knowledge production in the two fields, but to focus on journals with a high citation rate, and potentially exerting a tangible influence on their field. The sampling criteria was thus to select articles with strong legitimacy based on the academic prestige of the journals in which they were published [4].
The second step was selecting a sample of articles published within these 10 journals. Since the goal of our project was to study the patterns of knowledge circulation within medical education research—and higher education as a comparative field—we targeted research articles only, and excluded reviews, commentaries, letters, editorials, and other non-primary research formats as they do not report on empirical research.
The total number of research articles published in the 10 targeted journals during the year 2017 was 1046 (647 in medical education and 399 in higher education). Using a random number generator (random.org), we selected 10% of these articles, which totalled 64 in medical education and 41 in higher education for a total of 105 articles. Tables S1 and S2 outline the number of research articles published in each of the 10 targeted journals in 2017 and the number of articles we selected per journal. We have no reason to believe that the articles included in our sample are different from research articles not included.

The 105 articles included in our sample cited a total of 1959 articles: 1412 articles were referenced in the 64 medical education publications, and 547 articles were referenced in the 41 higher education publications. These 1959 referenced articles were distributed across 706 different journals: 447 different journals in medical education and 259 different journals in higher education. The lower number of journals (n=706) in comparison with the number of referenced articles (n=1959) means that some journals were cited by multiple referenced articles.

The procedure used to construct the dataset of referenced articles from the five most cited medical education journals and the five most cited higher education journals is shown in Fig. S1

Fig. S1 Procedure used to construct the dataset of referenced articles from the five most cited medical education journals and the five most cited higher education journals

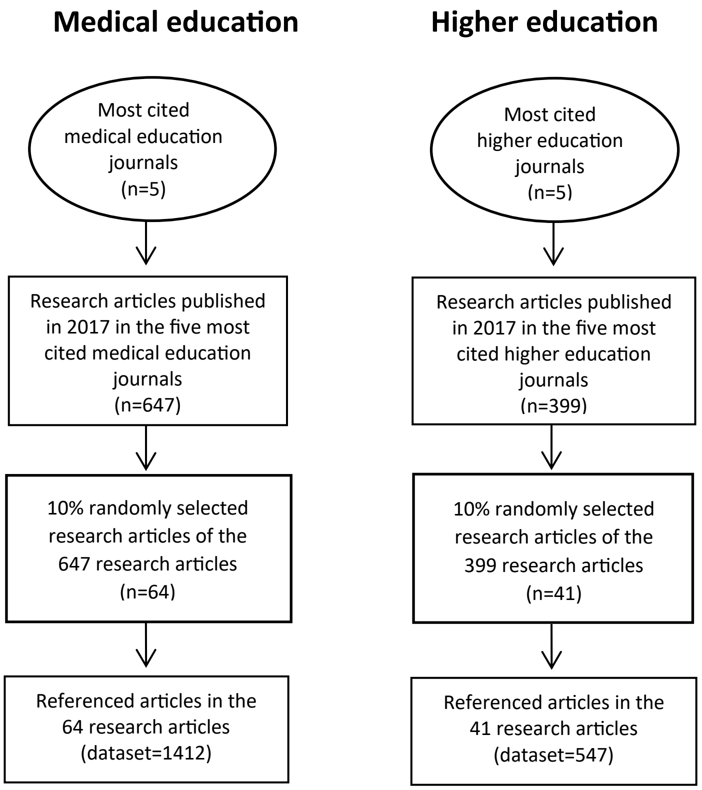

The same random sampling procedure was used for books and books chapters. The 64 medical education research articles referenced 154 books and 57 books chapters; 10% represents 15 books and six book chapters. The 41 higher education research articles referenced 373 books and 138 books chapters; 10% represents 37 books and 14 books chapters. In addition to the 1959 referenced articles, we included these 52 books and 20 books chapters for a total of 2031 references in our dataset. Table S3 outlines the distribution of research articles, books, and books chapters included in our dataset.

Table S3 Number of references analyzed in medical education and higher education research

	Articles	Books	Book Chapters	Total
Medical education	1412	15	6	1433
Higher education	547	37	14	598
Total	1959	52	20	2031

Data analysis

To analyze the references and identify which disciplines and research areas medical education and higher education researchers draw from, we inductively developed a typology of eight knowledge orientations, which we labelled knowledge clusters. These clusters are: 1) the Medical Education research knowledge cluster; 2) the Applied Health research knowledge cluster (mostly health services research/clinical research); 3) the Interdisciplinary Health research knowledge cluster; 4) the Disciplinary/Institutionalized research areas knowledge cluster; 5) the Education research knowledge cluster; 6) the Topic-Centred (non-health) research knowledge cluster; 7) the Profession/Science Education research knowledge cluster, and 8) the Higher Education research knowledge cluster. We decided to create a cluster for Education distinct from the Disciplines and Institutional research areas because one of the goals of our study was to be able to appreciate specifically the contribution of Education knowledge to the medical education research field.

The first step we took to develop these knowledge clusters was to examine the knowledge orientation of the journal in which the 1959 referenced articles where published. An effective way to do this is to turn to the 'Aims and Scope' of the journals. All journals have a web page dedicated to their 'Aims and Scope' where they outline their mandate and the type(s) of research they consider in alignment with their editorial orientation. The details provided typically list what disciplines, topics, research areas, and methods fall within their scope. In cases where the information posted was insufficiently detailed or unclear, members of the research team (MA and SL) read articles published in recent issues of the journals to ensure accurate categorization.

To conduct the categorization of the books and books chapters, we drew on all pertinent information available within each book and book chapter. The elements we typically considered were the title of the book and/or of the chapter, the table of contents, the foreword, the introduction, and any other parts of the document that could provide information about its content and knowledge orientation.

The eight inductively developed knowledge clusters served as our conceptual map to categorize the 2031 references in our dataset. For example, a reference coming from the *New England Journal of Medicine* (*NEJM*) was classified within the Applied Health Research knowledge cluster because *NEJM*, based on its aims and scope, is a journal whose primary research orientation is applied health research (in contrast, for example, to basic or disciplinary health research). In order to provide as much detail as possible on our categorization, we list in Table S4 the three journals with the highest number of citations for each cluster as well as one book and/or book chapter.

Since the material we are working with is inherently fluid, we chose to design our groupings of journals, books, and book chapters as 'clusters' instead of 'categories.' Clusters better reflect the fact that knowledge areas, topics, and disciplines have porous boundaries.

Table S4 Eight inductively developed knowledge clusters

Knowledge clusters	Medical education	Higher education
1. Disciplinary and Institutionalized research fields<br><br>Includes journals, books, books chap. focusing on disciplines (e.g. psychology, biology, sociology) or well-established research areas (e.g. business and management, organization studies, cognitive sciences).	3 most cited journals:<br>*Journal of Personality and Social Psychology; Qualitative Research in Psychology; Educational Psychologist.*<br>Representative book:<br>DelVecchio Good, MJ. (1995). *American Medicine: The quest for competence*.	3 most cited journals:<br>*American Political Science Review; Economics of Education Review; American Journal of Political Science.*<br>Representative book:<br>Dewey, J. (2001). *Democracy and Education: An Introduction to the philosophy of education*.

2. Topic Centred (non health)<br><br>Includes journals, books, books chap. focusing on a specific topic, but not health related (e.g. accident prevention, industrial ergonomic, migration and human security).	3 most cited journals:<br>*Educational and Psychological Measurement; Assessment; Journal of Vocational Behavior.*<br>Representative book:<br>Crotty, M. (1988). *The foundations of social research: Meaning and perspective in the research process*.	3 most cited journals:<br>*Qualitative Enquiry; Journal of Education and Work; Journal of College Student Development.*<br>Representative book:<br>Stronach, I., & MacLure, M. (1997). *Educational research undone: The postmodern embrace*.
3. Education<br><br>Includes journals, books, books chap. focusing on education research.	3 most cited journals:<br>*Review of Educational Research; Journal of the Learning Sciences; Theory into Practice.*<br>Representative book:<br>No book or book chapter cited.	3 most cited journals:<br>*Review of Educational Research; British Educational Research Journal; Learning and Instruction.*<br>Representative book:<br>Lave, J., & Wenger, E. (1991). *Situated Learning: Legitimate Peripheral Participation*.
4. Medical Education<br><br>Includes journals, books, books chap. focusing on any aspects of medical education.	3 most cited journals:<br>*Academic Medicine; Medical Education; Medical Teacher.*<br>Representative book chapter:<br>White CB, et al. (2010). Self-regulation learning in medical education. In: Swanwick T, ed., *Understanding medical education*.	3 most cited journals:<br>No journal cited.<br>Representative book:<br>No book or book chapter cited.
5. Interdisciplinary Health<br><br>Includes interdisciplinary journals, books, books chap. focusing on health-related issues.	3 most cited journals:<br>*PLoS ONE; Social Science and Medicine; Simulation in Healthcare.*<br>Representative book:<br>Koenig HG, et al. (2012). *Handbook of religion and health.*	3 most cited journals:<br>*Child Development; PLoS ONE* (only two journals cited)<br>Representative book:<br>No book or book chapter cited.
6. Applied Health Research (mainly health services research and clinical research)<br><br>Includes journals, books, books chap. focusing on applied health research.	3 most cited journals:<br>*Journal of the American Medical Association; Journal of General Internal Medicine; New England Journal of Medicine.*<br>Representative book:<br>No book or book chapter cited.	3 most cited journals:<br>No journal cited.<br><br>Representative book:<br>No book or book chapter cited.
7. Profession / Science Education<br><br>Includes journals, books, books chap. focusing on all aspects of professional and scientific education.	3 most cited journals:<br>*Academy of Management Learning and Education; Art Education; Education for Information.*<br>Representative book:<br>No book or book chapter cited.	3 most cited journals:<br>*Journal of Geography in Higher Education; CBE-Life Sciences Education; Journal of Education for Business.*<br>Representative book:<br>No book or book chapter cited.
8. Higher education<br><br>Includes journals, books, books chap. focusing on all aspects of higher education.	3 most cited journals:<br>*Planning for Higher Education; CDN Journal of Higher Education; Internet and Higher Education*<br>Representative book:	3 most cited journals:<br>*Studies in Higher Education; Higher Education Research & Development; Research in Higher Education.*<br>Representative book:

	No book or book chapter cited.	Palfreyman, D., & Tapper, T. (2014). *Reshaping the university: The rise of the regulated market in higher education*.

References

1. Jacobs JA. In Defense of Disciplines Interdisciplinarity and Specialization in the Research University. Chicago: University of Chicago Press; 2014.
2. Porter AA, Rafols I. Is science becoming more interdisciplinary? Measuring and mapping six research fields over time. Scientometrics. 2009;81:719-45.
3. Gingras Y. Bibliometrics and research evaluation. Uses and abuses. Cambridge MA: MIT Press; 2014.
4. Chen S, Arsenault C, Gingras Y, et al. Exploring the interdisciplinary evolution of a discipline: The case of Biochemistry and Molecular Biology. Scientometrics. 2015;102:1307-23.
